# Cigarette and Cannabis Smoking Effects on GPR15+ Helper T Cell Levels in Peripheral Blood: Relationships with Epigenetic Biomarkers

**DOI:** 10.3390/genes11020149

**Published:** 2020-01-30

**Authors:** Allan M. Andersen, Man-Kit Lei, Steven R. H. Beach, Robert A. Philibert, Sushmita Sinha, John D. Colgan

**Affiliations:** 1 Department of Psychiatry, University of Iowa, Iowa City, IA 52242, USA; robert-philibert@uiowa.edu; 2 Department of Sociology, University of Georgia, Athens, GA 30602, USA; karlo@uga.edu; 3 Center for Family Research, University of Georgia, Athens, GA 30602, USA; srhbeach@uga.edu; 4 Department of Psychology, University of Georgia, Athens, GA 30602, USA; 5 Behavioral Diagnostics, Coralville, IA 52241, USA; 6 Department of Pathology, University of Iowa, Iowa City, IA 52242, USA; sushmita-sinha@uiowa.edu; 7 Department of Internal Medicine, University of Iowa, Iowa City, IA 52242, USA; john-colgan@uiowa.edu; 8 Department of Anatomy and Cell Biology, University of Iowa, Iowa City, IA 52242, USA

**Keywords:** biomarkers, epigenetics, smoking, tobacco, cannabis, digital PCR

## Abstract

**Background**: Smoking causes widespread epigenetic changes that have been linked with an increased risk of smoking-associated diseases and elevated mortality. Of particular interest are changes in the level of T cells expressing G-protein-coupled receptor 15 (GPR15), a chemokine receptor linked with multiple autoimmune diseases, including inflammatory bowel disease, multiple sclerosis and psoriasis. Accordingly, a better understanding of the mechanisms by which smoking influences variation in the GPR15+ helper T cell subpopulation is of potential interest. **Methods**: In the current study, we used flow cytometry and digital PCR assays to measure the GPR15+CD3+CD4+ populations in peripheral blood from a cohort of n = 62 primarily African American young adults (aged 27–35 years) with a high rate of tobacco and cannabis use. **Results**: We demonstrated that self-reported tobacco and cannabis smoking predict GPR15^+^CD3^+^CD4^+^ helper T cell levels using linear regression models. Further, we demonstrated that methylation of two candidate CpGs, cg19859270, located in *GPR15*, and cg05575921, located in the gene *Aryl Hydrocarbon Receptor Repressor* (*AHRR*), were both significant predictors of GPR15^+^CD3^+^CD4^+^ cell levels, mediating the relationship between smoking habits and increases in GPR15^+^CD3^+^CD4^+^ cells. As hypothesized, the interaction between cg05575921 and cg19859270 was also significant, indicating that low cg05575921 methylation was more strongly predictive of GPR15^+^CD3^+^CD4^+^ cell levels for those who also had lower cg19859270 methylation. **Conclusions**: Smoking leads changes in two CpGs, cg05575921 and cg19859270, that mediate 38.5% of the relationship between tobacco and cannabis smoking and increased GPR15^+^ T_h_ levels in this sample. The impact of cg19859270 in amplifying the association between cg05575921 and increased GPR15^+^ T_h_ levels is of potential theoretical interest given the possibility that it reflects a permissive interaction between different parts of the adaptive immune system.

## 1. Introduction

Smoking is the leading cause of preventable morbidity and mortality in the United States, and is responsible for nearly half a million deaths per year [1,2]. Chronic illnesses with inflammatory components, including cancer [3], chronic obstructive pulmonary disease (COPD), cardiovascular disease (CVD), and stroke [4] contribute to the majority of deaths caused by smoking. Smokers also suffer from elevated rates of autoimmune diseases including Crohn’s disease [5], rheumatoid arthritis [6], and multiple sclerosis [7], as well as elevated rates of inflammatory skin and gut disorders [8,9]. Unfortunately, the pathophysiologic mechanisms underlying smoking’s relationships to these diseases are not yet well understood. One approach to better understanding these mechanisms is to identify mediators of the effects of smoking on key systemic regulators known to influence disease outcomes. In particular, effects mediated by smoking’s well-established effects on DNA methylation are promising.

In the current investigation, we chose to focus on an understudied systemic effect of smoking, i.e., its impact on levels of CD3+CD4+ helper T (T_h_) cells expressing the G-protein-coupled receptor 15 (GPR15), a chemokine receptor encoded by the chromosome 3 gene *GPR15*. Originally identified as a coreceptor for simian immunodeficiency virus (SIV), human immunodeficiency virus 2 (HIV-2), and some human immunodeficiency virus 1 (HIV-1) strains [10,11,12], GPR15 has been linked to multiple smoking-associated diseases, including rheumatoid arthritis [13], inflammatory bowel disease [14,15,16], and multiple sclerosis [17], and psoriasis [18]. 

Recently, interest in the relationship between smoking and GPR15^+^ T_h_ cells has increased as a result of multiple epigenome-wide studies of studies demonstrating associations between smoking and hypomethylation of cg19859270, a CpG located in the first exon of *GPR15* [19,20,21,22,23]. Bauer and colleagues [24,25] provided insight into this association by demonstrating two findings. First, smokers had a highly significant (*p* = 1.8 × 10 ^−10^) increase in the peripheral CD3^+^ T cells expressing GPR15 (15.5% in smokers vs. 3.7% in non-smokers), and to a lesser extent B cells, and second, these GPR15^+^CD3^+^ T cells had a markedly lower average methylation of cg19859270 than GPR15^−^CD3^+^ T cells, resulting in the arithmetic difference in mean methylation at cg19859270 observed in whole blood. 

Interestingly, smoking-associated hypomethylation of cg19859270 appears to be more pronounced in individuals of African Ancestry. In one study of 972 African Americans [21], cg19859270 was the second most highly smoking-associated locus in the epigenome, while in two other African American cohorts cg19859270 was the first and second most strongly associated probe, respectively [26,27]. In contrast, cg19859270 was not among the top 25 smoking-associated CpGs in a large meta-analysis of smoking-associated CpG sites in which the majority (76%) of included individuals were of European Ancestry [28]. One reason for these discrepant results may be *cis* genetic variants that moderate methylation status at cg19859270 and whose frequencies are ethnically contextual, such as rs2230344 (minor allele frequency in African Americans is 0.06; in Europeans, 0.23) [27]. 

A second methylation locus of potential relevance in understanding smoking-associated changes in GPR15^+^ T_h_ cell levels is cg05575921, located in the chromosome 6 gene *Aryl Hydrocarbon Receptor Repressor* (*AHRR*). In addition to being perhaps the most well-replicated finding in environmental epigenetics [23,28,29,30], smoking-associated hypomethylation of cg05575921 is a highly sensitive and specific indicator of smoking status, with Receiver Operating Characteristic (ROC) areas under the curve (AUC) as high as 0.99 reported [22,31,32]. Underlying cg05575921’s strong performance as a biomarker, as well as the reliability of its association with smoking, is its large “delta beta”, i.e., the magnitude of difference in methylation in smokers compared to non-smokers. Whereas most smoking-associated CpGs demonstrate delta betas of less than 10%, in heavy smokers methylation at cg05575921 may be 50% or more below the population average of around 85%–90% [30]. This large dynamic range also facilitates the measurement of dose-response effects [28,33,34,35] and reversibility of cg05575921 hypomethylation associated with smoking cessation [36,37,38,39]. 

Smoking-associated changes in methylation at cg05575921 may also provide insight into biological responses to smoking. Specifically, the methylation status of it is thought to influence expression of *AHRR* [22], a key regulator of the xenobiotic pathway responsible for detoxification of polyaromatic hydrocarbons found in tobacco and cannabis smoke [40] that has also been shown to influence inflammatory responses and act as a tumor suppressor gene in several types of cancers [41]. Cg05575921 hypomethylation has also been directly linked to elevated systemic inflammation, as indicated by serum C-reactive protein (CRP) [42], and an increase in overall mortality risk [43,44]. Lastly, in contrast to cg19859270, smoking-associated hypomethylation of cg05575921 is not influenced by genetic background, providing additional utility as a tool for investigating smoking’s biological effects in populations of mixed ancestry [28]. Lastly, in contrast to cg19859270, hypomethylation of cg05575921 has been shown in multiple studies to primarily occur in granulocytes and monocytes, suggesting a distinct role for these cell populations in biological responses to smoking [45,46]. 

Although concern that GPR15+ T cells may be drivers of chronic inflammatory disease processes in smokers [47], their physiological role and the exact nature of their relationship to smoking remain unclear. Kim [48] found that mice deficient in GPR15 developed severe large intestinal inflammation, suggesting a potentially protective role in immune homeostasis, while Bauer and colleagues [49] found that the level of GPR15-expressing T cells was unrelated to the lung disease status in human smokers and non-smokers. Bauer and colleagues [45] also found that smoking was associated with increased GPR15 expression across a broad range of T cell subtypes in adults, suggesting GPR15+ T cells may be adaptive rather than pathogenic. 

In addition to questions as to the therapeutic value of interventions targeting GPR15+ T cells in human smokers at risk for inflammatory disease, relationships between smoking patterns, GPR15 expression, and other immunological variables remain unclear. In particular, the differential impact of tobacco vs. cannabis smoking patterns on GPR15 expression in T cells has not been previously explored but is of potential significance given the anti-inflammatory effects of some cannabinoids [50]. In examining this relationship, other immunological variables of potential impact include psychological factors influencing the HPA axis [51], adiposity [52], non-steroidal anti-inflammatory drug (NSAID) use [53], and ancestry. The latter factor may be of particular relevance both because of the strong link between smoking and hypomethylation of cg19859270 in African Americans, reviewed above, and from a public health perspective because African Americans bear a disproportionate burden of smoking-associated illnesses [54,55] and demonstrate higher levels of systemic inflammation than European American smokers [56]. 

Lastly, it is unknown whether the net effect of the above factors on variance in T cells expressing GPR15 is fully accounted for by changes in methylation at cg19859270 and cg05575921, respectively indicating the transcriptional status of GPR15 and systemic exposure to smoke, and whether interactions between the methylation status of these two CpGs can account for additional variance in this cell population. 

Here, we examine those relationships using novel digital PCR methods in a cohort of African Americans young adults with a high rate of smoking, testing the following hypotheses:

1. First, we examine whether the proportion of GPR15-expressing CD3+CD4+ T_h_ cells is associated with (a) tobacco and cannabis smoking patterns and (b) confounding variables that could impact immune function such as age, sex, race, perceived stress, depressive symptomatology, and use of over-the-counter non-steroidal anti-inflammatory drugs (NSAIDs).
2. Second, we examine whether the addition of cg19859270 and cg05575921 can fully account for the variance in the level of GPR15-expressing CD3^+^CD4^+^ T_h_ cells due to smoking patterns and other immunological variables.
3. Third, we examine whether interaction effects between cg19859270 and cg05575921 influence the level of GPR15-expressing CD3^+^CD4^+^ T_h_ cells.

## 2. Materials and Methods 

### 2.1. Sample

The Family and Community Health Study (FACHS) is a mulita-site investigation of neighborhood and family effects on child health and development, consisting of several hundred African American families living in Georgia and Iowa. Each family who participated in the FACHS study included at least one child subject between the ages of 10 and 12 years old at the time of recruitment (1995–1997). Child subjects have been characterized over seven waves of data collection since the beginning of the study. Later waves of FACHS have also included subjects’ romantic partners, who along with the subjects themselves consented to be recontacted for future studies. A more detailed description of the FAHCS sample and methods for the study is available elsewhere [57,58,59]. 

### 2.2. Subject Procedures

Participants in the current study included FACHS subjects (now adults in their early 30s) and romantic partners, currently living in Iowa who participated in the last wave of the study (wave seven, collected 2014–2016), and had agreed to be recontacted for future studies. Study subjects were recruited by phone by a trained research assistant and provided written consent to participate. 

Subjects were administered a brief, structured interview on their use of tobacco products, cannabis products, and other nicotine-containing products, including e-cigarettes. Next, to allow for analyses controlling for common factors that may impact immune function, subjects then completed three structured self-report forms. First, they were asked to report on their average consumption of over-the-counter NSAIDs. Second, subjects completed a nine-item Patient Health Questionnaire depression module (PHQ-9) [60], and third, a Perceived Stress Scale [61]. Subjects then provided self-reported data on their ethnicity (African American or other), age, height, and weight for calculation of body-mass index (BMI) as a proxy for adiposity, the latter due its potential impact on immune function [62]. 

All study protocols and procedures were approved by the Institutional Review Board at the University of Iowa (Title: Tobacco and Cannabis Smoking Effects on Immune Function in Youth; IRB ID # 201604748). 

### 2.3. Biomaterials and Assays

Following completion of data collection, subjects were phlebotomized to provide whole blood for DNA preparation and flow cytometry assays, and sera, according to our previously published protocols [31,63]. 

#### 2.3.1. ELISAs

Serum cotinine and tetrahydrocannabinol (THC) levels were assayed by enzyme-linked immunoassay (ELISA) with kits supplied by Abnova (Taiwan) as previously described [31]. 

#### 2.3.2. ddPCR Assays

Determination of the methylation status at cg05575921 and cg19859270 was conducted using ddPCR implementation of the previously described quantitative PCR approach [26]. First, 1 μg of DNA from each subject was bisulfite converted using an EpiTect Fast 96 DNA Bisulfite kit (Qiagen, Germany) according to the manufacturer’s direction. 

The methylation ratio at cg05575921 (C/(C+T)) in each bisulfite treated sample was then determined using the Smoke Signature^®^ Assay (IBI Scientific, Peosta, IA, USA) and a QX200 Droplet Digital PCR System™ (Bio-Rad, Hercules, CA, USA) according to the manufacturer’s protocols. In brief, an aliquot of the bisulfite-converted DNA was pre-amplified with the Smoke Signature® Pre-Amp Master Mix under high stringency conditions per the manufacturer’s protocol, and then diluted between 1:1000 and 1:5000. Then, 5 μL of the resulting solution was mixed with 1.1 μL of 20X Smoke Signature Assay, 6.5 μL of water, and 11 μL of BioRad 2X ddPCR Supermix (no dUTP), and vortexed. 

The resulting mixture was then processed with a Bio Rad Automated Droplet Generator, which generated approximately 20,000 micelles, with each containing approximately 1 nanoliter of PCR mixture, and PCR amplified (95 °C × 10′, then 40 cycles of 95 °C × 15″ and 55 °C × 60″, and finally 98 °C × 10′). After amplification was complete, the post-amplification allele content status (either C, T, C+T, or blank) of each micelle was determined by a QX200 Droplet Reader and the percent methylation status of each sample calculated using BioRad’s QuantaSoft software (v1.7) (BioRad: Hercules, CA, USA). The methylation ratio at cg19859270 was similarly determined using a custom-designed primer and probe ddPCR assay for the sequence surrounding the CpG, following identical procedures to those described for cg05575921. Primer and probe sequences are available in File S5. 

#### 2.3.3. PBMC Isolation and Cryopreservation

Peripheral blood mononuclear cells (PBMCs) were isolated using Vacutainer CPT tubes from Becton Dickinson (BD; Franklin Lakes, NJ, USA) according to the manufacturer’s protocol. Immediately following isolation, PBMCs were cryopreserved in liquid nitrogen in a solution of 10% DMSO, 20% fetal calf serum, 70% RPMI supplemented with HEPES (1M, pH range 7.2–7.5) and L-glutamine via slow temperature-lowering method with a Mr. Frosty Nalgene polyethylene vial holder (Thermo Fisher Scientific, San Jose, CA, USA) containing isopropyl alcohol. 

Cells were stored in liquid nitrogen for >= 1 week before thawing. Prior to staining, PBMCs were rapidly thawed in a solution of 10% fetal calf serum, 90% RPMI supplemented with DNAse in a 37 °C water bath. Samples were washed in FACS buffer (1X PBS containing 1% BSA and 0.1% sodium azide). Viability and recovery were measured using tryptan blue exclusion. 

#### 2.3.4. Flow Cytometry

Following washing in FACS buffer, PBMCs were stained with anti-CD3 (clone UCHT1, BD), anti-CD4 (monoclonal, clone Sk3, BD), and either anti-GPR15 (clone SA302A10, BioLegend, San Diego, CA, USA)) or a mouse IgG2a kappa isotype control (clone MOPC-2710, BioLegend). Samples were resuspended in 100 μL of FACS buffer for staining and were stained with 1 μL of each antibody as per the manufacturer’s recommendations. Samples were acquired on a Becton Dickinson LSR II flow cytometer using FACS Diva Software. Fully stained PBMCs were compared with unstained samples and samples stained with IgG2a kappa isotype control. The percent of CD3^+^CD4^+^ T cells staining positive for GPR15 (hereafter “GPR15^+^ T_h_ cell percent”) for each subject was visualized and calculated using FlowJo software version 7 (Tree Star, San Carlos, CA, USA). 

### 2.4. Statistical Analysis

Statistical analyses were conducted with the R [64] statistical software. The complete dataset used for the analyses below is included as Table S6.

#### 2.4.1. Coding

Self-report data on tobacco, cannabis, and nicotine-containing products were coded as follows. Subjects who endorsed any use of combusted tobacco or cannabis products within a given time frame (week, month, year, or ever) were coded as positive for a derived variable reflecting any smoke inhalation over that time period. Similarly, subjects who endorsed any use of nicotine-containing products, including tobacco products and e-cigarettes, were coded as positive for nicotine use over that time period, and subjects who endorsed any use of cannabis were coded positive for cannabis use over the same time period. 

Intensity of cannabis use was coded based on subjects’ self-reported use of cannabis over the past year. Due to high variability in how subjects reported their use of cannabis, units of one “blunt” and one “joint” were each coded as equal a 0.5 g dose of cannabis. Cumulative consumption of cannabis in grams over the last year was then coded on a 0 to 2 scale, with 0 reflecting no use in the past year, 1 less than 50 g consume in the past year, and 2 reflecting 50 g or more consumed in the past year. For tobacco use, due to the infrequency of use of tobacco products other than cigarettes, intensity was coded as the numeric average cigarettes per day consumed. 

Perceived Stress Scale and PHQ-9 total scores were calculated according to published instructions. Subjects reported their dose and frequency use of three NSAIDs: ibuprofen, aspirin, and naproxen, which were then converted to equipotent doses of ibuprofen 200 mg [65]. 

Serum cotinine levels of 1 ng/mL or greater were coded as positive for the purposes of analysis. Similarly, serum THC levels of 0.5 ng/mL or greater were coded as positive. An additional variable was derived reflecting positivity for either cotinine or THC or being negative for both. 

#### 2.4.2. Correlational Analyses

Correlational analyses were used to examine the relationship between self-reported smoking of both tobacco and cannabis, methylation of cg05575921 and cg19859270, and other subject characteristics using the function rcorr(). The same analyses were then repeated including only subjects concordant for any self-reported smoking (tobacco and/or cannabis) and any serum positivity (cotinine and/or THC). Visualization of correlations between study variables was done using the corrplot() function in R.

#### 2.4.3. ANOVAs

The significance of differences in mean levels of methylation at cg05575921, cg19859270, and GPR15^+^ T_h_ cell percent between groups were measured by ANOVA using the aov() function. Post hoc testing was done by Tukey’s method using the TukeyHSD() function. 

#### 2.4.4. Regression Analyses

Multiple regression analyses were used to examine the effect of self-reported smoking intensity of tobacco and/or cannabis on the GPR15^+^ T_h_ cell percent, with and without covariates that could potentially impact immune cell function and proliferation. Of note, we use the term “prediction” in to refer to linear predictors in the context of multiple regression modeling, rather than to indicate validation of these models in a second dataset. 

To examine methylation at cg05575921 and cg19859270 as potential mediators of the association between self-reported smoking and GPR15^+^ T_h_ cell percent, the two CpGs were entered in conjunction with the other predictors to examine whether these methylation signatures could account for the impact of smoking on GPR15^+^ T_h_ cell percent, reducing the observed effect of self-reported smoking and confounders to non-significance. Then, to explore the potential for interaction between the two CpGs to account for additional variance in GPR15^+^ T_h_ cell percent, methylation values for each CpG were centered and normalized, with the interaction of the resulting variables then entered as an additional predictor in the regression model. 

The above models were then repeated on a subset of subjects demonstrating concordance for tobacco or cannabis smoking (self-report and serum positivity) or non-use (self-report and serum negativity) to more stringently examine the above hypotheses. 

Next, to examine the specific effects of tobacco and cannabis smoking on GPR15^+^ T_h_ cell percent, dummy variables tobacco-only smoke exposure and cannabis-only smoke exposure in the last year were added to the above models, Subjects were coded as “tobacco-only” if they were positive for self-reported tobacco smoking (cigarette or cigar) in the last year, or demonstrated serum cotinine positivity, and were negative for both self-reported cannabis smoking and serum THC. Similarly, “cannabis-only” subjects had endorsed cannabis smoking or demonstrated serum THC positivity but could not have self-reported any tobacco smoking in the last year or were positive for serum cotinine.

Lastly, to examine the relative contributions of cg05575921, cg19859270, and their interaction term as predictors of GPR15^+^ T_h_ cell percent, the three predictors were entered into a linear regression model which was then analyzed using the Relative Importance of Regressors in Linear Models package, relaimpo(), [66] using the method of partitioning R^2^ by averaging over orders (“lmg”).

## 3. Results

### 3.1. Subject Characteristics and Assay ResuLts

Contact information was available for a total of 169 FACHS young adult subjects living in Iowa who had agreed to be recontacted for future studies. Of these, 81 were contacted and agreed to participate in the study. 

Subject demographic characteristics, clinical variables, and laboratory results and their correlations are summarized in Table 1. Consistent with the nature of the cohort, a longitudinal study of subjects enrolled in 5th grade starting in 1996, there was a tight age distribution around 31 years. The majority of subjects were African Americans, with a preponderance of females. Notably, subjects had a high rate of obesity, with the majority of body mass index (BMI) values calculated at over thirty. Most subjects rated their level of depressive symptomatology between the minimal and moderate ranges [60], while average stress levels were reported as higher than the population average [61]. Sixty percent of subjects reported smoking cigarettes at some point in their lives, while 77% reported at least one use of cannabis. Serum enzyme-linked immunoassays (ELISAs) of cotinine and tetrahydrocannabinol (THC) were completed for 71 subjects. 

A total of 65 subjects had both cg05575921 and cg19859270 digital droplet PCR (ddPCR) assays completed. Two subjects failed quality control for the cg19859270 ddPCR assay, defined as greater than a 5% confidence interval for the methylation value, while no subjects failed the cg05575921 assay, leaving a total of 63 subjects with both ddPCR assays available for analysis. Lastly, flow cytometry experiments to measure the percent of CD3^+^CD4^+^ T cells staining positive for GPR15 (hereafter “GPR15^+^ T_h_ cell percent”) were completed for 62 of those 63 subjects, leaving a total of 62 subjects for whom all measures were completed. 

### 3.2. Study Variable Correlations

**Figure 1:**
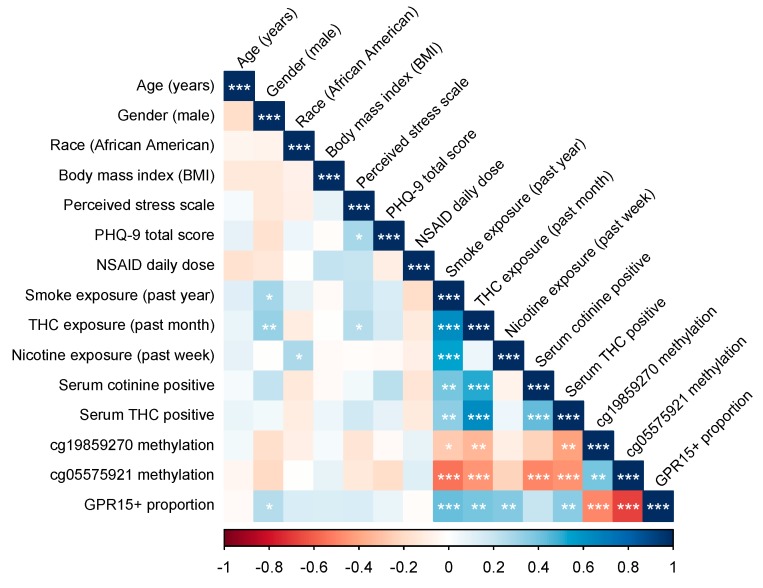
Correlations between study variables analyzed. BMI refers to body mass index. Perceived Stress Scale refers to the Perceived Stress Scale total score. PHQ-9 refers to the Patient Health Questionnaire depression module total score. Nonsteroidal anti-inflammatory drug (NSAID) daily dose refers to 200 mg equivalent doses of ibuprofen, naproxen, and aspirin. Smoke exposure indicates tobacco and/or cannabis smoking. Serum cotinine positive indicates serum values of > 1 ng/mL, while serum tetrahydrocannabinol (THC) positive indicates > 0.5 ng/mL. GPR15+ proportion refers to the proportion of CD3+CD4+ PBMCs. Significance codes: * *p* ≤ 0.05; ** *p* ≤ 0.01; *** *p* ≤ 0.001.

Correlations between study variables are depicted in Figure 1 and are available in Table S1. Results of serum ELISA assays for cotinine and THC indicated an overall high rate of current cannabis use (32%) and nicotine-containing products (31%), with 44% of subjects positive for one or both. 

Interestingly, subject self-reports of cannabis use were strongly associated with serum THC positivity (r = 0.631, *p* = 3.86 × 10 ^−8^), whereas prior-week use of nicotine containing products was uncorrelated with serum cotinine positivity (r = −0.062, *p* = 0.630). This may reflect differences in THC and cotinine persistence, cotinine assay problems, patterns of episodic use in this sample, or differences in accuracy of self-reporting. To control for the latter possibility, primary linear regression analyses were rerun using only participants with biochemically confirmed use.

Digital PCR assays for cg19859270 and cg05575921 indicated a range of methylation values similar to other studies with a mix of smokers and non-smokers [25,37], with cg05575921 demonstrating the greater dynamic range, as previously reported [30]. Methylation values at the two CpGs were moderately correlated with each other (R = 0.395, *p* = 0.00149). Both CpGs were correlated with self-reported tobacco smoking, cannabis smoking, and serum THC positivity, while serum cotinine positivity was strongly correlated with cg05575921 methylation but only showed a trend association with cg19859270, as depicted in Figure 1 and detailed in Table S1. 

**Figure 2:**
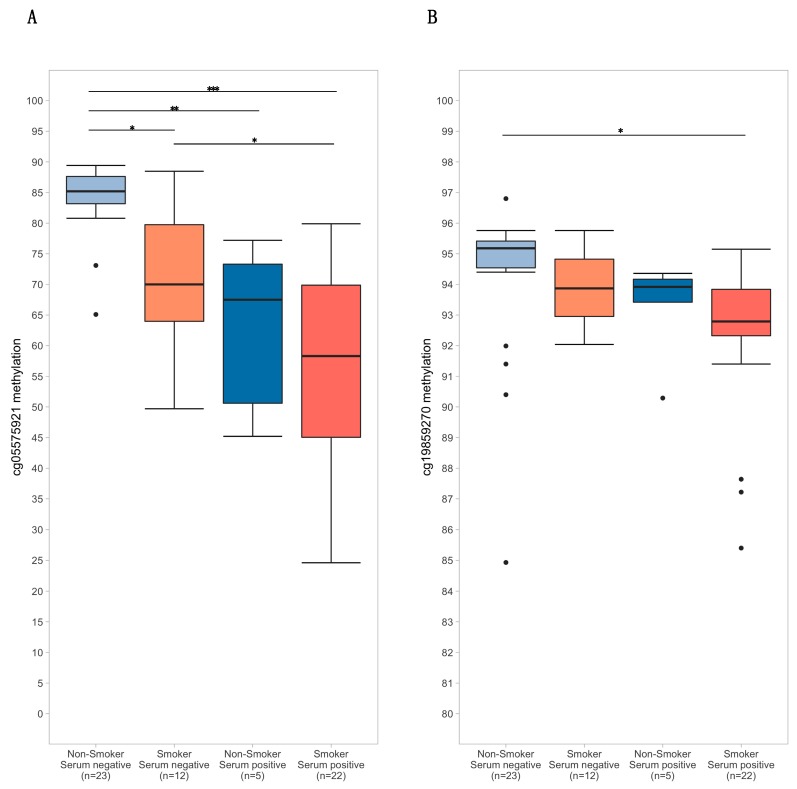
Boxplots of cg05575921 (**A**) and cg19859270 (**B**) methylation stratified by self-reported smoking status (tobacco and/or cannabis) and serum positivity (cotinine > 1 ng/mL and/or tetrahydrocannabinol (THC) > 0.5 ng/mL). Significance codes: * *p* ≤ 0.05; ** *p* ≤ 0.01; *** *p* ≤ 0.001 (Tukey’s Honest Significant Difference).

The distribution of methylation values for cg05575921 and cg19859270, conditioned on (1) self-reported smoking of either tobacco or cannabis products in the last year and (2) serum positivity for either cotinine or THC, is depicted in Figure 2. 

**Figure 3:**
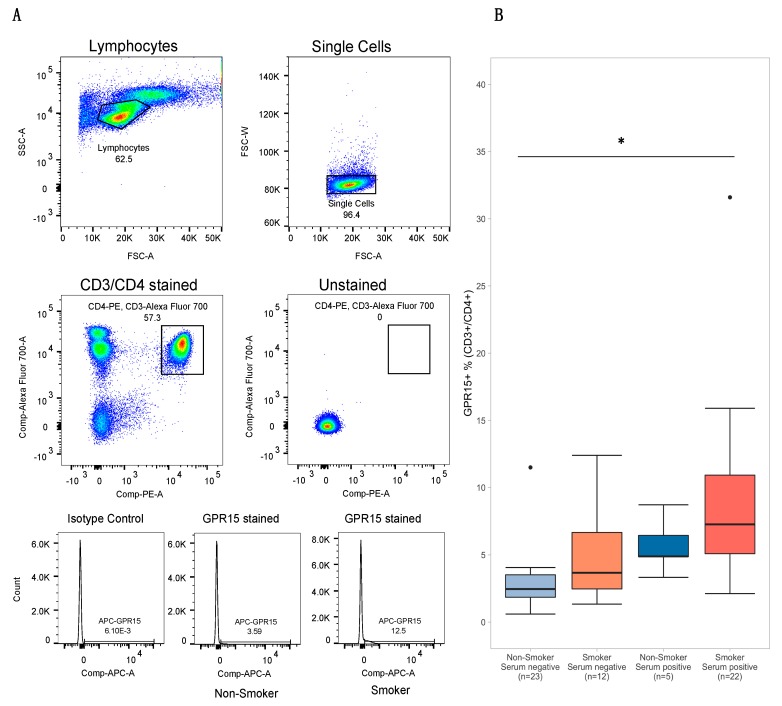
GPR15+ Th cell flow cytometry gating strategy and results stratified by smoking status and serum positivity. Panel (**A**) depicts the flow cytometry gating strategy used to visualize GPR15^+^ T_h_ cell percent within the CD3^+^CD4^+^ population of peripheral lymphocytes. Histograms at bottom depict GPR15^+^ T_h_ cell percent data from two subjects, one a “non-smoker”, negative for both self-reported smoking and serum cotinine and tetrahydrocannabinol (THC) , and the other a “smoker”, positive for self-reported smoking and both cotinine and THC. The leftmost histogram depicts the results from the same “non-smoker” using the mouse IgG2a kappa isotype as a negative control. Panel (**B**) depicts boxplots of T_h_ cell percent for subjects stratified by self-reported smoking status (tobacco and/or cannabis) and serum positivity (cotinine > 1 ng/mL and/or THC > 0.5 ng/mL). Significance codes: * *p* ≤ 0.05; ** *p* ≤ 0.01; *** *p* ≤ 0.001 (Tukey’s Honest Significant Difference).

The flow cytometry gating strategy used to measure GPR15+ Th cell percent is depicted in Figure 3A. Across all subjects, GPR15^+^ T_h_ cell percent ranged from 0.60 to 31.60, with a mean of 5.71 (*SD* 5.04). Subjects positive for cotinine or THC had a mean GPR15^+^ T_h_ cell percent of 8.33 (*SD* 5.92) with a range of 2.12 to 31.6, while those negative for both cotinine and THC had a mean of 3.67 (*SD* 3.00) and a range of 0.60 to 12.4. GPR15^+^ T_h_ cell percent was significantly correlated with male gender, self-reported smoking in the past year, cannabis smoking in the past month, nicotine use in past week, serum THC status, and both cg05575921 and cg19859270, as shown in Figure 1 and Table S1, but not with other subject characteristics. 

For comparison, a correlation table for the study variables including only the *n* = 45 subjects concordant for self-reported smoking (tobacco and/or cannabis) and serum indicators (serum cotinine and/or THC) was constructed, otherwise including the same variables as in Table S1. As seen in Table S2, characteristics of subjects who were concordant for self-reported use versus non-use of cigarettes and cannabis were similar to the group as a whole, with the exception that correlations between epigenetic and serum biomarkers were strengthened. 

### 3.3. Regression Analysis Results

Multiple linear regression models were used to examine the relationships between smoking behavior, DNA methylation, changes in GPR15^+^ T_h_ cell percent, with and without covariates that could potentially affect immune function and proliferation, and also to examine mediational hypotheses. 

First, GPR15^+^ T_h_ cell percent was regressed against two variables reflecting self-reported intensity of cigarette and cannabis smoking, respectively, as shown in Table 2. The first model (Model 1A) demonstrated that both cigarettes per day and scaled cannabis use over the last year were significant predictors of GPR15^+^ T_h_ cell percent with an R^2^ of 0.357 (*p* = 4.18 × 10^−7^). The addition of predictors potentially impacting immune composition and function (Model 1B) did not further improve model fit. In contrast, the subsequent addition of cg05575921 and cg19859270 (Model 1C) did improve the fit (R^2^ = 0.590, *p* = 1.52 × 10^−8^). Interestingly, while both CpGs were significant predictors of GPR15^+^ T_h_ cell percent in Model 1C, cigarettes per day, though not average cannabis use, remained a significant predictor (*p* < 0.01), indicating that the impact of intensity of self-reported cigarette use was not fully mediated by the two candidate CpGs. Lastly, the interaction term for cg05575921 and cg19859270 was introduced (Model 1D), yielding a significant effect (*p* < 0.001) and improved model fit (R^2^ = 0.670, *p* = 2.21 × 10^−10^), while cigarettes per day remained a significant predictor (*p* < 0.01) of GPR15^+^ T_h_ cell percent. Although BMI was a significant (*p* = 0.05) predictor of GPR15^+^ T_h_ cell percent in models 1C and 1D, a post-hoc linear regression analysis including BMI as a sole predictor did not show a significant relationship with GPR15^+^ T_h_ cell percent (*p* = 0.282). Mediation analysis of Models 1B-1D using the simple path model equation indicated that the two CpGs mediated 35.51% of the variance in GPR15^+^ T_h_ cell percent due to cigarettes per day, and 38.53% with the addition of their interaction term.

To more stringently examine the above relationships, equivalent models were run restricting to the n = 45 subjects demonstrating concordance between self-reported smoking intensity (tobacco and/or cannabis) and serum indicators (serum cotinine and/or THC). As shown in Table S3, the full model incorporating both CpGs and their interaction term as predictors (Model 2D) demonstrated the best overall fit, with an R^2^ of 0.779 (*p* = 1.83 × 10^−9^). Similar to the above results, the interaction term between the two CpGs was a significant predictor (*p* < 0.001) and improved the significance of cg19859270 as a predictor (*p* < 0.05), while cg05575921 (*p* = 5.30 × 10^−5^) and cigarettes per day (*p* < 0.01) also remained significant predictors of GPR15^+^ T_h_ cell percent. 

A third set of regression analyses were performed to explore specific effects of tobacco vs. cannabis smoking on GPR15^+^ T_h_ cell percent. As shown in Table S4, the addition of dummy variables indicating tobacco-only smoking (n = 10 subjects) and cannabis-only smoking (*n* = 9 subjects) in the last year did not improve model fit compared to Model 1A (Model 3A, *R*^2^ = 0.370, *p* = 3.50 × 10^−6^), nor did the addition of immunologic variables (Model 3B, *R*^2^ = 0.344, *p* = 4.16 × 10^−4^). The addition of the two CpGs as predictors did improve fit (Model 3C, *R*^2^ = 0.585, *p* = 7.38 × 10^−8^), as did the addition of their interaction term (Model 3D, *R*^2^ = 0.666, *p* = 1.35 × 10^−9^), similar to models 1C and 1D. Variables indicating tobacco-only and cannabis-only smoking were not significant predictors of GPR15^+^ T_h_ cell percent in any of the above models, whereas cigarettes per day but not average cannabis use remained a significant predictor in the full model (*p* < 0.05). Similarly, although the tobacco-only subjects had a higher mean GPR15^+^ T_h_ cell percent (7.3, SD 4.4) than the cannabis-only smokers (4.6, 3.0), the difference was not significant (*p* = 0.13), nor did the two groups demonstrate significantly different mean levels of cg05575921 methylation (*p* = 1) or cg19859270 methylation (*p* = 1). 

**Figure 4:**
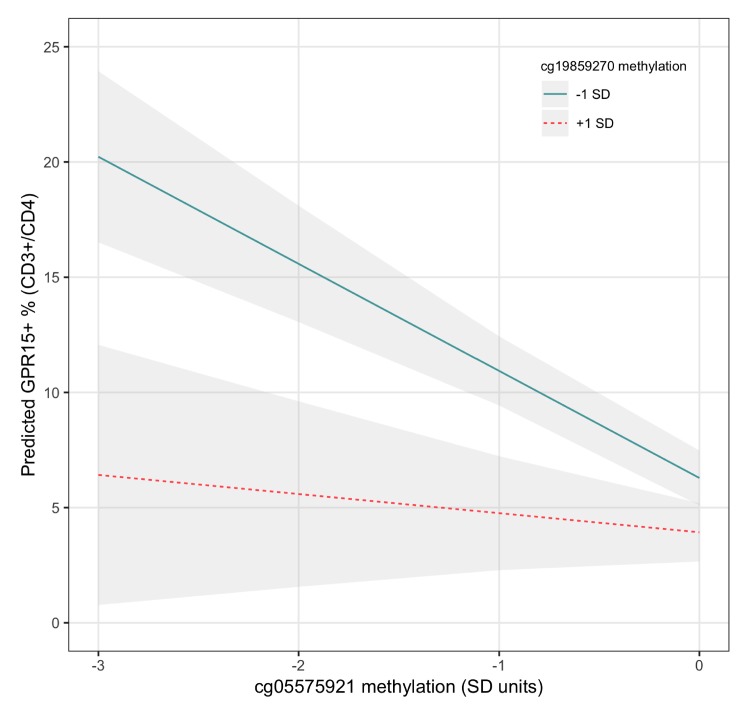
Interaction of cg05575921 and cg19859270 methylation in predicting GPR15^+^ T_h_ cell percent. The interaction is visualized by plotting regression lines for cg05575921 while holding the value of cg19859270 at 1 SD above and below its mean. Regression coefficient values are taken from Model 2D, Table S3. The ribbons indicate the model-based 95% confidence intervals for each regression line.

Given the consistent findings that methylation of cg19859270 interacted with cg05575921 to predict GPR15^+^ T_h_ cell percent, this interaction was plotted as the effect of high (+1 *SD)* versus low (-1 *SD*) cg19859270 methylation values on the relationship between cg05575921 methylation and GPR15^+^ T_h_ cell percent, as depicted in Figure 4. As demonstrated in the figure and by the corresponding regression model (Model 2D), low cg19859270 methylation intensified the effect of decreasing cg05575921 methylation, reflective of increasing smoke exposure, on expansion of the GPR15^+^ population of CD3^+^CD4^+^ T_h_ cells.

Lastly, to examine the relative contributions of cg05575921, cg19859270, and their interaction term as linear predictors of GPR15^+^ T_h_ cell percent, the three terms were entered into a linear regression model which was then analyzed using the relaimpo() package in R using the default settings, *n* = 1000 bootstraps. The overall model was significant (*R*^2^ = 0.583, *p* = 1.09 × 10 ^−11^), as was each predictor individually. The relative contributions of cg05575921 was greatest at 36.2% (95% CI 23.4%–36.2%), followed by cg19859270 at 14.9% (2.4%–36.2%), and their interaction term at 9.3% (0% to 22.7%). The same procedure was then performed using the full model (Model 1D) including smoking intensity variables and immunologic predictors in addition to the two CpGs and their interaction term. Results of that analysis were similar, showing a relative contribution of cg05575921 of 27.9% (15.5%–44.3%), cg19859270 of 11.5% (2.9%–30.0%), and their interaction term of 7.4% (0%–16.1%). The contribution of cigarettes per day was 13.8% (4.1%–27.7%) and scaled marijuana use was 4.8% (1.4%–11.6%), while the remaining predictors all contributed less than 3% of the variance individually. 

## 4. Discussion

Smoking is associated with increased morbidity and mortality. Although the mechanisms underlying this risk are not fully understood, smoking-associated increases in GPR15-expressing immune cells are implicated in a number of illnesses that affect smokers. However, their biological role and mechanisms underlying their strong association with smoking are not well understood. 

In this study, we examined a cohort of young African Americans, over half of whom reported lifetime exposure to tobacco and cannabis smoke, exploring differential effects of tobacco and cannabis smoking on GPR15^+^ T_h_ cell percent in the context of other immunological variables and epigenetic indicators of smoking, using a series of multiple linear regression models. We confirmed the association between increased GPR15^+^ T_h_ cell percent and smoking habits found by Bauer and others [25,49] finding independent effects of cigarette and cannabis smoking intensity in our preliminary model, suggesting additive effects of tobacco and cannabis smoke exposure. However, after accounting for other immunological variables, the significant main effect of cannabis smoke exposure was reduced to non-significance. This finding was confirmed by supplemental analyses including analyses focused on biochemically confirmed tobacco-only smokers, cannabis-only smokers, and non-smoking controls.

The addition of epigenetic indicators of smoking, i.e., decreased methylation at cg05575921 and cg19859270, substantially improved prediction of GPR15^+^ T_h_ cell percent in the context of our regression models, consistent with our hypotheses that these two methylation loci reflect both systemic exposure to smoke and the transcriptional status of *GPR15*, but did not fully account for self-reported smoking’s association with GPR15^+^ T_h_ cell percent, with cigarettes per day remaining a significant predictor. This finding suggests that smoking intensity influences GPR15^+^ T_h_ cell percent through mechanisms not fully captured by the methylation state of two CpGs or other variables measured. This finding is consistent with prior work by Andersen and colleagues [32], who demonstrated that self-report of smoking habits, when accurate, performed approximately equally as well as measurement of cg05575921 methylation in predicting serum cotinine status, but that the combination of both self-report and cg05575921 methylation was overall most predictive. 

We hypothesized that ancestry, particularly African American ancestry, might influence the relationship between smoking and GPR15^+^ T_h_ cell percent. In particular, Dogan and colleagues [27] previously demonstrated that rs2230344, the only common non-synonymous SNP in the immediate region of cg19859270, showed a significant main effect on the relationship between smoking status and cg19859270 methylation in African Americans, though not in European Americans. In our study, we were unable to genotype subjects to confirm this effect but did not find an effect of ancestry when entered in conjunction with other predictors. It is unclear whether the ascertainment of rs2230344 genotype in addition to methylation status at cg05575921 and cg19859270 would greatly improve the ability to predict GPR15 levels, although this hypothesis could be readily tested in future investigations.

Although a minor effect of BMI on GPR15^+^ T_h_ cell percent was seen in Model 1C and 1D, the effect was not present prior to the introduction of the two CpGs and their interaction term to the regression model, nor was it seen in models restricted to subjects concordant for self-reported smoking habits and serum indicators of smoking. This inconsistent finding could be an artifact of our relatively small sample size, or might be the result of negative correlations among the predictors, but nonetheless suggests that controlling for BMI may be important in future studies examining the relationship between smoking, GPR15^+^ T_h_ levels, and inflammatory disease, particularly given prior reports linking obesity with elevated of C-reactive protein [67,68]. Other potentially confounding demographic and behavioral variables that could affect immune composition and function did not significantly contribute to GPR15^+^ T_h_ cell percent, although larger studies in the future could uncover more subtle effects. 

The finding that greater hypomethylation of cg19859270 amplified the relationship between smoke exposure, as indicated by cg05575921 hypomethylation, and GPR15^+^ T_h_ cell percent is consistent with expectations that decreased methylation of exonic CpGs facilitates greater gene expression. The mechanism underlying significant interaction between cg05575921 and cg19859270 is not clear but may be influenced by several factors. Because hypomethylation of cg05575921 and cg19859270 in whole blood have been shown to be linked with more pronounced hypomethylation in distinct immune cell populations, specifically monocytes and granulocytes (cg05575921) and T and B cells (cg19859270) [24,46], one possibility is that the effect observed may therefore indicate unmeasured interactions between these cell populations. 

In this context, it should be noted that methylation of the promoter region of *methylene tetrahydrofolate reductase* (*MTHFR*) was recently shown to influence smoking-induced hypomethylation of both cg05575921 [69] and cg19859270 [70]. In fact, cg19859270 was the locus most highly influenced by *MTHFR* promoter methylation across the genome (*p* = 9.04 × 10 ^−15^) in the study of Andersen and colleagues [32]. This suggests the availability of donor methyl groups for DNA methylation, DNA replication, and other cellular processes may be an additional unmeasured factor in our study influencing GPR15^+^ T_h_ cell percent. 

Although they have been associated with a variety of inflammatory conditions, the biological role of GPR15+ expressing T cells is not clear. Bauer [45] and colleagues recently demonstrated that GPR15 expression was not specific to any T cell subtype, although it was most expressed on T_h_17 cells, and suggested a potentially protective role for GPR15-expressing T cells in chronic smoking. Going forward, further mechanistic studies are needed to establish whether GPR15-expressing T cells convey risk, benefit, or neither in smokers and whether they may be targets for therapeutic interventions. 

If relevant to health, measurement of GPR15+ T cell levels directly by flow cytometry may be the most appropriate in clinical settings. In contrast, accurate estimation of the GPR15+ T cell levels by epigenetic assays requires analysis of a large number of CpGs to accurately estimate cell proportions [71]. While this information may be obtained through the use of a genome-wide chip such as the Illumina 450k, this assay is costlier and time consuming than flow cytometry. The custom designed digital PCR probes used in this study, by comparison, are significantly less costly (we estimate under $10 in reagents per sample), but do not provide cell proportion data. However, the use of such probes may be useful in large-scale studies where DNA but not peripheral blood samples suitable for flow cytometry are available, and the cost of genome-wide array-based assays may be prohibitive. 

Several unexpected findings in our study are worth noting. First, we observed that a number of our subjects with the greatest GPR15^+^ T_h_ cell percent did not demonstrate serum cotinine positivity. Examination of our dataset (available in Table S5) shows that most of these subjects were either positive for serum THC or self-reported recent tobacco or cannabis smoking. Similarly, a single individual who was negative for self-report of both tobacco and cannabis use, and negative for serum cotinine and THC had a GPR15^+^ T_h_ cell percent above 5%. However, examination of this data point showed evidence of hypomethylation of cg05575921, consistent with chronic smoke exposure. Lastly, it is interesting that the subject with the most hypomethylation of cg19859270 was in the serum negative, self-report negative group, and showed a cg05575921 methylation value within the non-smoking range (88%). Taken together these findings highlight the utility of obtaining complementary sources of information regarding substance use habits and the potential for assay failure.

Because the relatively small sample size examined in the current study is a limitation, replication in an independent sample is needed to confirm whether the combination of digital PCR assays for cg05575921 and cg19859270 truly improves prediction of GPR15^+^ T_h_ cell percent. The small sample size may have also obscured other significant contributions to GPR15^+^ T_h_ cell percent such as cannabis-specific smoking patterns, and ethnicity, particularly the low number of non-African Americans in our sample. Second, as discussed above, because we were unable to control for cell type proportions in our analyses, caution is warranted in interpretation of our results, and future experiments with cell proportion data are needed to confirm our findings. Third, the small amount of variability of cg19859270 compared to cg05575921 or direct measurement of GPR15^+^ T_h_ cell percent suggest that it has little utility as an independent biomarker for smoking. Fourth, a low rate of cotinine positivity in the subjects with the greatest GPR15^+^ T_h_ cell percent suggests that poor ELISA assay performance may have impacted our results, despite strong correlations between cotinine positivity and cg05575921 methylation. 

## 5. Conclusions

In conclusion, we have demonstrated that two smoking-associated CpGs, cg05575921 and cg19859270, and their interaction, mediate a substantial portion (38.5%) of the relationship between tobacco smoking and increased GPR15^+^ T_h_ levels in the context of multiple linear regression models. In addition, the impact of cg05575921 demethylation on GPR15+Th levels was amplified by cg19859270, suggesting the possibility of enhanced prediction of cell level using only these two smoking-associated CpGs. The impact of cg19859270 in amplifying the association between cg05575921and increased GPR15^+^ T_h_ levels is of potential theoretical interest given the possibility that it reflects a permissive interaction between different parts of the adaptive immune system. Because GPR15^+^ T_h_ levels have suggestive links with smoking-associated diseases, further efforts to define its biological role in both smokers and non-smokers are needed. Our results suggest that it may be feasible to estimate GPR15^+^ T_h_ cell levels in settings where direct measurement of GPR15^+^ T_h_ cells are not possible, such as large DNA biobanks. 

## Figures and Tables

**Table 1:** Means and standard deviations for study variables, all subjects (*n* = 62).

Variable	M	SD
1. Age (years)	30.7	1.2
2. Gender (male)	0.37	-
3. Race (African American)	0.86	-
4. Body mass index (BMI)	34.0	8.5
5. Perceived Stress Scale	19.3	4.4
6. PHQ-9 total	4.7	4.1
7. NSAID daily dose	0.43	1.00
8. Any smoking (past year)	0.55	0.50
9. Cannabis use (past month)	0.32	0.47
10. Nicotine use (past week)	0.31	0.47
11. Cotinine positivity	0.31	0.47
12. THC positivity	0.32	0.47
13. cg19859270 methylation	93.4	2.4
14. cg05575921 methylation	70.3	16.7
15. GPR15+ % (CD3+/CD4+)	5.7	5.0

BMI refers to body mass index. Perceived stress scale refers to the Perceived Stress Scale total score. PHQ-9 refers to the Patient Health Questionnaire depression module total score. Nonsteroidal anti-inflammatory drug (NSAID) daily dose refers to 200 mg equivalent doses of ibuprofen, naproxen, and aspirin. Any smoking indicates exposure to tobacco and/or cannabis smoke. Cotinine positivity indicates serum values of > 1 ng/mL, while tetrahydrocannabinol (THC) positivity indicates > 0.5 ng/mL.

**Table 2:** Multiple linear regression results for GPR15+ Th cell percent regressed against tobacco and cannabis smoking intensity.

Model	Model 1A		Model 1B		Model 1C		Model 1D	
Parameters	b	*p*-Value	b	*p*-Value	b	*p*-Value	b	*p*-Value
Cigarettes per day	0.588	**6.33 × 10^−5^**	0.597	**1.48 × 10^−4^**	0.385	**2.56 × 10^−3^**	0.367	**1.47 × 10^−3^**
Scaled cannabis use	1.80	**1.00 × 10^−2^**	1.18	1.38 **× 10^−1^**	0.138	8.33 **× 10^−1^**	0.198	7.36 **× 10^−1^**
Age (years)			0.014	9.76 **× 10^−1^**	0.0365	9.20 **× 10^−1^**	0.0940	7.74 **× 10^−1^**
Gender (male)			2.47	**4.51 × 10^−2^**	1.58	1.08 **× 10^−1^**	1.38	1.18 **× 10^−1^**
Race (African American)			1.08	4.83 **× 10^−1^**	1.31	2.89 **× 10^−1^**	1.01	3.62 **× 10^−1^**
BMI			0.0766	2.40 **× 10^−1^**	0.105	**4.57 × 10^−2^**	0.0973	**3.99 × 10^−2^**
Perceived Stress Scale			0.0422	7.56 **× 10^−1^**	0.0152	8.89 **× 10^−1^**	−0.0424	6.68 **× 10^−1^**
PHQ-9 total			0.145	3.21 **× 10^−1^**	0.0518	6.59 **× 10^−1^**	0.165	1.38 **× 10^−1^**
NSAID daily dose			0.0939	8.69 **× 10^−1^**	0.218	6.31 **× 10^−1^**	0.211	6.05 **× 10^−1^**
cg19859270 methylation					−0.420	**3.74 × 10^−2^**	−0.480	**9.13 × 10^−3^**
cg05575921 methylation					−0.139	**4.70 × 10^−5^**	−0.115	**2.10 × 10^−4^**
cg19859270*cg05575921							1.44	**6.67 × 10^−4^**
Constant	3.65	4.18 **× 10^−7^**	−2.42	8.72 **× 10^−1^**	46.9	3.23 **× 10^−2^**	49.6	1.23 **× 10^−2^**
R-square	0.357		0.348		0.590		0.670	
*p*-value	8.21 **× 10^−7^**		1.68 **× 10^−4^**		1.52 **× 10^−8^**		2.21 **× 10^−10^**	

Cigarettes per day calculated by average consumption over the past month. Scaled cannabis use indicates average use over the past year. BMI refers to body mass index. Perceived Stress Scale refers to the Perceived Stress Scale total score. PHQ-9 refers to the Patient Health Questionnaire depression module total score. Nonsteroidal anti-inflammatory drug (NSAID) daily dose refers to 200 mg equivalent doses of ibuprofen, naproxen, and aspirin. Cotinine positivity indicates serum values of > 1 ng/mL, while THC positivity indicates > 0.5 ng/mL. GPR15+ proportion refers to the proportion of CD3+CD4+ PBMCs. Regression results shown for all subjects (*n* = 62). Note: the cg19859270*cg05575921 was centered and normalized prior to entry into the regression model.

## Supplementary Materials

The following are available online at https://www.mdpi.com/2073-4425/11/2/149/s1. Table S1. Correlations: means, and standard deviations for study variables, all subjects (*n* = 62), Table S2. Correlations, means, and standard deviations for study variables, restricting to cotinine/THC verified subjects (*n* = 45), Table S3. Multiple linear regression results for GPR15+ Th cell percent regressed against tobacco and cannabis smoking intensity, restricting to cotinine/THC verified subjects (*n* = 45). Smoking intensity indicated by cigarettes per day and a scaled average of cannabis use over the past year. Additional predictors include cg05575921 methylation, cg19859270 methylation, a cg05575921*cg19859270 interaction term, and demographic and behavioral covariates regressed on GPR15+ Th cell percent. Note: the cg19859270*cg05575921 was centered and normalized prior to entry into the regression model, Table S4. Multiple linear regression results for GPR15+ Th cell percent regressed against tobacco and cannabis smoking intensity with dummy variables indicating tobacco-only smoking (*n* = 10) and cannabis-only smoking (*n* = 9). Smoking intensity indicated by cigarettes per day and a scaled average of cannabis use over the past year. Additional predictors include cg05575921 methylation, cg19859270 methylation, a cg05575921*cg19859270 interaction term, and demographic and behavioral covariates. Note: the cg19859270*cg05575921 was centered and normalized prior to entry into the regression model, File S6. Cg19859270 digital PCR assay primer and probe sequences, and Table S6. Dataset used for all analyses including biological assays, self-report data, demographic variables and behavioral measures.
