# Supplementary material for: Cigarette and Cannabis Smoking Effects on GPR15+ Helper T Cell Levels in Peripheral Blood: Relationships with Epigenetic Biomarkers
Source: Genes (Basel). 2020 Jan 30;11(2):149. doi: 10.3390/genes11020149 (PMC7074551; doi:10.3390/genes11020149)
Supplement: Supplementary file 1 [file genes-11-00149-s001.zip › Supplements revised/File S5.docx]

C target Sequence: ATG AGT GTT GAT CGT TAT TTG GTT ATT GTG TGG TTA GTC GTA TTT AGG AAA TTT AGA AGG ATA GAT TGT GTA TAT GTA GTT TGT GT

T target Sequence: ATG AGT GTT GAT CGT TAT TTG GTT ATT GTG TGG TTA GTT GTA TTT AGG AAA TTT AGA AGG ATA GAT TGT GTA TAT GTA GTT TGT GT

Forward primer: ATGAGTGTTGATCGTTATTTGGTTAT

Reverse primer: ACACAAACTACATATACACAATCTATCC

C allele probe: /5HEX/ TA+C+G+ACTAA+C+CA

T allele probe: /56-FAM/ ATA+C+A+A+CTAA+C+CAC
